# The effect of compaction pressure and sintering temperature on phase evolution and technological properties of alumina mullite zirconia ceramics

**DOI:** 10.1038/s41598-026-60881-4

**Published:** 2026-07-16

**Authors:** M. M. S. Wahsh, T. S. Mansour

**Affiliations:** Refractories, Ceramics and Building Materials Department, National Research Centre, 12622, Elbuhouth St., Dokki, Cairo, Egypt

**Keywords:** Sintering, Ceramic composites, X-ray diffraction, Microstructure, Engineering, Materials science

## Abstract

Alumina–mullite–zirconia ceramic composites were fabricated using a solid-state reaction involving nano silica, alumina, and zirconia powder mixes at varying sintering temperatures (1450–1600 °C) and compaction pressures (80–160 MPa). Magnesia was added in a consistent proportion to stabilize the tetragonal phase of zirconia at room temperature and as sintering aid. The densification parameters of sintered ceramic composites, namely bulk density, apparent porosity, and linear change, were measured. The phase composition and microstructure of sintered ceramics were determined using XRD and SEM. The results show that a specific quantity of nano silica (5 wt%) plays an important role in improving the sintering and densification parameters of sintered samples. Compaction pressures of up to 120 MPa enhanced the densification parameters in sintered samples due to increases in powder compact and sold state sintering processes. Stress-induced phase transformation from tetragonal to monoclinic in zirconia, which is accompanied by a 3–5% volume expansion, may improve the mechanical properties of sintered samples. This volume expansion forms a compressive stress zone around a crack tip, thereby preventing it from propagating.

## Introduction

Advanced ceramics are widely used across numerous fields because their composition and microstructural characteristics can be tailored to achieve specific performance requirements^1^. Alumina (Al_2_O_3_) and zirconia (ZrO_2_), especially in the form of yttria-stabilized tetragonal zirconia polycrystals (Y-TZP)^2–4^, are two of the most widely used materials. These materials are also widely used in composites like alumina-toughened zirconia (ATZ) and zirconia-toughened alumina (ZTA)^5^. These ceramics are appropriate for harsh environments due to their excellent mechanical strength, electrical functioning, thermal stability, and optical characteristics. They are therefore used in industries including energy systems, aircraft, refractory technologies (such as burners, filters, and engine components), electronic devices (such as semiconductors, capacitors, and substrates), and biomedical applications (such as implants and dental components)^1–5^. Industrial production of alumina- and zirconia-based ceramics typically relies on established forming techniques, including powder compaction and colloidal processing routes^3,6^.

In fine-grained alumina, deformation at elevated temperatures is caused by rapid grain growth, which occurs under both tension and compression conditions. Adding a second-phase zirconia to reduce grain growth and increase alumina’s high-temperature ductility is one way to mitigate this effect^7^. However, in several instances, these tactics have demonstrated only modest efficacy. Zirconia is still extensively used in alumina matrices because it contributes to increased mechanical performance via transformation toughening. Stress-induced change from tetragonal to monoclinic phase in zirconia particles at crack tips absorbs energy and slows crack propagation^8^.

The use of certain dopants, which aid in controlling grain size during sintering and minimise residual stresses resulting from thermal expansion mismatch, can further enhance ATZ systems^9–12^. Ceramics with three distinct phases exhibit better high-temperature ductility than two-phase systems^13^. An example is the alumina–mullite–zirconia (AMZ) composite, in which mullite and zirconia are distributed within an alumina matrix. These secondary phases act to restrict abnormal grain growth in alumina and thereby contribute to improved mechanical performance^14,15^. In mechanical engineering, power generation, and glass manufacture, alumina-based materials are widely utilized in parts including nozzles, tubes, plungers, and feeders^16,17^. In such applications, key objectives include maximizing fracture toughness, hardness, strength, elastic modulus, wear resistance^18–21^, and oxidation resistance^22^. It has been demonstrated that adding mullite and zirconia improves mechanical integrity and thermal shock resistance. Mechanisms such crack bridging, compressive stress generation, and phase transformation toughening are responsible for the significant increases in fracture toughness^7^.

The strengthening of alumina-zirconia- silica composites is achieved through a multi-phases approach that combines intrinsic phase transformations with extrinsic microstructural barriers. At the core of this system is transformation toughening, a mechanism primarily driven by the zirconia component^23^. In its stable state at room temperature, zirconia is often held in a metastable tetragonal phase through the use of stabilizers. When the material is subjected to mechanical stress specifically at the tip of a developing crack the energy is sufficient to trigger a martensitic transformation from the tetragonal phase to the monoclinic phase^24^. This transition is crucial because it results in a localized volume expansion of approximately 3% to 5%^25^. This expansion generates a " compressive stresses " around the crack tip, effectively acting as internal pressure that clamps the crack shut and prevents further propagation^23^. Complementing this phase transformation are the roles played by alumina and silica in managing crack energy. Alumina serves as a high-modulus reinforcement that promotes crack deflection and crack bridging^25^. Because alumina grains are exceptionally hard and rigid, they force a moving crack to take a more tortuous, zigzag path around the grains rather than moving in a straight line. This increases the total energy required for fracture by extending the crack’s path length. Furthermore, grains that span across the crack faces can act as bridges, exerting a cohesive force that resists the separation of the crack walls^25^. The silica component, often present as a secondary phase or part of a complex silicate like mullite, introduces an additional strengthening layer through residual stress management^26^. By carefully controlling the composition, engineers can exploit the differences in the coefficient of thermal expansion (CTE) between the various phases. During the cooling process after sintering, the mismatch between the silica-rich phases and the zirconia-alumina matrix can induce significant compressive stresses on the surface of the material^27^.

The sintering of alumina-zirconia-silica ceramic composites involves complex thermo-chemical interactions that transition the material from a loose powder to a dense, multi-phase solid. This process is driven by the reduction of surface energy and is characterized by concurrent solid-state and liquid-phase reactions^28^. The introduction of silica (SiO_2_) into the alumina-zirconia matrix significantly alters the sintering kinetics by promoting liquid phase sintering^28^. At elevated temperatures, silica can form a silicate glass phase or interact with alumina to create an alumina-rich melt^28,29^. This liquid phase serves multiple roles: it provides a capillary force that pulls particles together, acts as a lubricant to reduce interparticle friction, and facilitates rapid mass transport via diffusion through the liquid medium^30^. This often allows for a reduction in the required sintering temperature compared to pure oxide systems^28,29^. As sintering progresses, a critical chemical reaction occurs between the alumina and silica components known as mullitization^31,32^. Alumina and silica react in situ to form mullite (3Al_2_O_3_•2SiO_2_), resulting in an alumina-zirconia-mullite composite^26,28^. During the final cooling stage of sintering, the different Coefficients of Thermal Expansion (CTE) and Young’s moduli of alumina, zirconia, and mullite create internal stresses^26^. Specifically, if the silica-based phase (like mullite) has a lower CTE than the substrate, it can generate compressive residual stresses throughout the ceramic^26^. These stresses are beneficial as they reinforce the material against crack propagation^26^. Zirconia particles are usually distributed at the grain boundaries of the alumina or mullite matrix in the microstructure, which is a dense network^26,33^. The presence of zirconia serves a mechanical purpose during the sintering process by acting as a grain growth inhibitor^30^. Zirconia particles, which do not easily dissolve into the alumina matrix, pin the grain boundaries of the alumina^30^. This pinning effect prevents excessive grain coarsening, leading to a more uniform and refined microstructure, which is essential for maintaining the high strength of the final ceramic^30^.

Recently, several studies have been conducted on the Al_2_O_3_-ZrO_2_-SiO_2_ system. Zhou et al. (2026) created SiO_2_-Al_2_O_3_-ZrO_2_ ternary ceramic cores to overcome the limitations of conventional silica- and alumina-based cores, particularly their low strength and refractoriness^34^. The results showed that interactions between silica, alumina, and zirconia led to strong interfacial bonding and microstructural pinning effects, which significantly improved high-temperature deformation resistance. The composition of 90% silica, 4% alumina, and 6% zirconia, when sintered at 1200 °C, exhibited flexural strength of 7.9 MPa at room-temperature, a high-temperature strength of 31.3 MPa at 1550 °C, a linear shrinkage of 1.18%, and a creep deflection of only 0.21 mm, demonstrating excellent comprehensive properties suitable for advanced investment casting applications^34^. Sun et al. (2026) also investigated microstructural control mechanisms in zirconia-modified alumina ceramics using molecular dynamics simulations, theoretical analysis, and experimental confirmation^35^. The microstructural regulation mechanisms in zirconia-modified alumina ceramics by combining molecular dynamics simulations, theoretical analysis, and experimental validation. It demonstrated that the biphasic Al_2_O_3_–ZrO_2_ system develops an interlocking microstructure that restricts atomic movement, effectively suppressing grain growth and stabilizing phases, with crystallite growth reduced by up to 43%. The analysis also revealed a unique structural evolution where zirconia preferentially segregates to the surfaces of alumina grains, forming a compact interfacial layer that results in a laminated “Al_2_O_3_-ZrO_2_ interfacial layer-Al_2_O_3_” configuration. This behavior extends to the material’s pores and cracks, where zirconium-rich phases accumulate to create “bridging traces” that enhance the material’s structural integrity through crack-bridging^35^.

Various processing routes have been developed for producing alumina-mullite-zirconia composites. These include conventional sintering of mixed powders (alumina, silica, and zirconia), slip casting using suspensions containing alumina, mullite, and zircon, and sol-gel techniques in which mullite precursors are first synthesized from alkoxide solutions, followed by the incorporation of alumina and zirconia to form the final composite structure^36–40^.

**Fig. 1:**
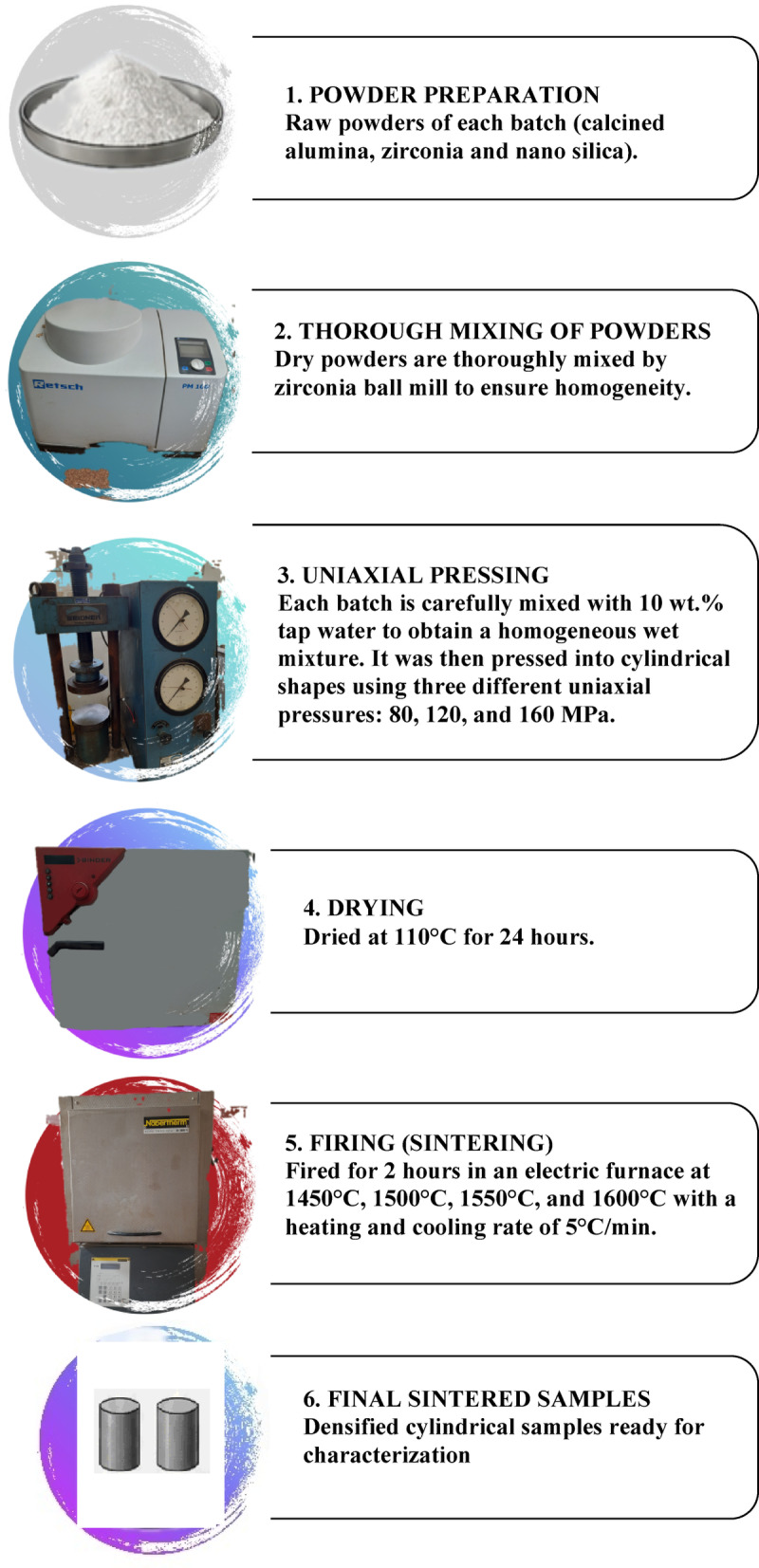
Process flow diagram for the preparation method.

The aim of this study is to investigate the effects of processing parameters such as compaction pressure, sintering temperature, and nano silica additions on phase composition, including in-situ mullite formation and microstructure, as well as the technological properties of sintered alumina-mullite-zirconia ceramics. The process flow diagram for the preparation method is shown in Fig. 1.

## Materials and experimental methods

### Starting materials

**Fig. 2:**
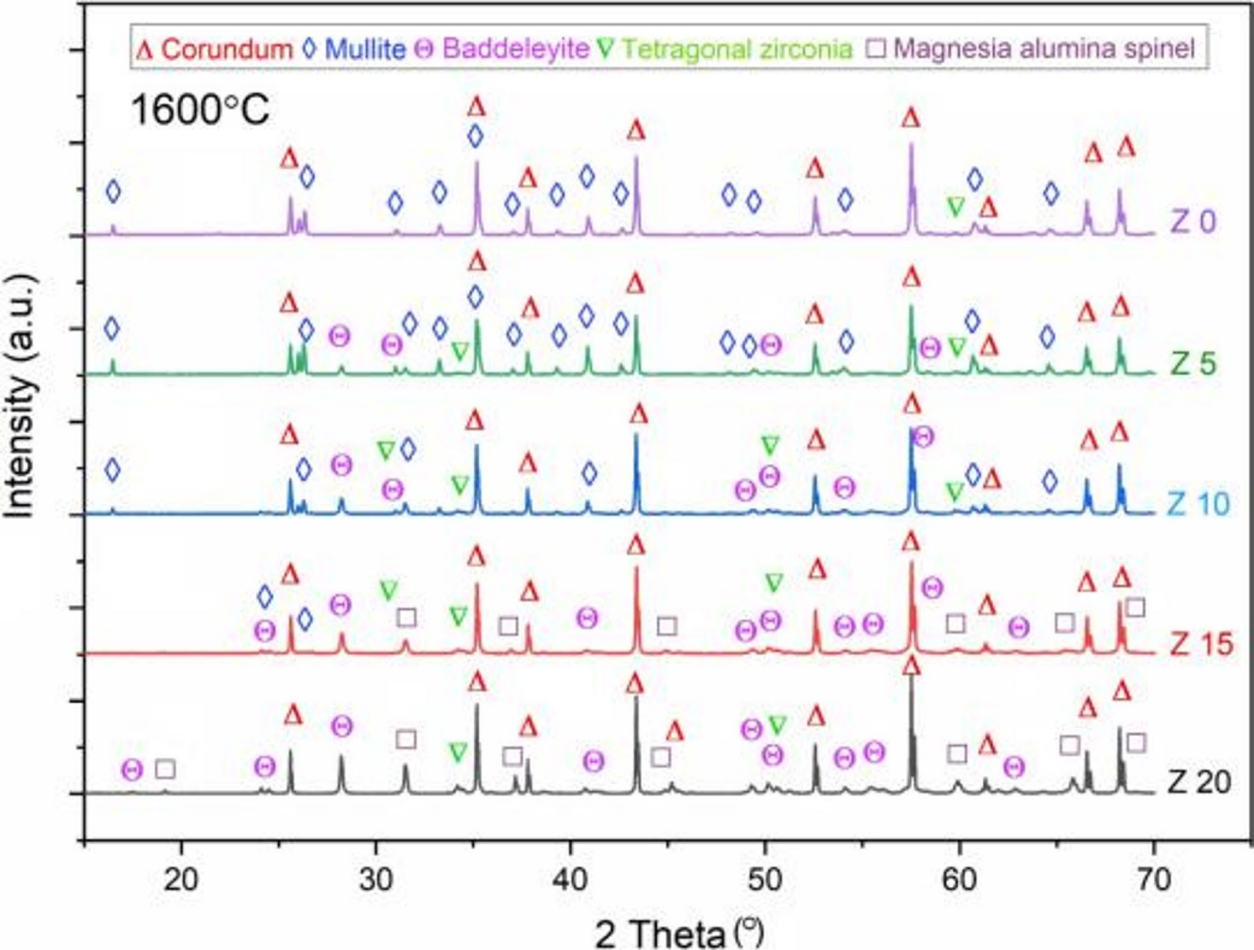
XRD patterns of sintered samples (Z0-Z20) at 1600 °C.

**Fig. 3:**
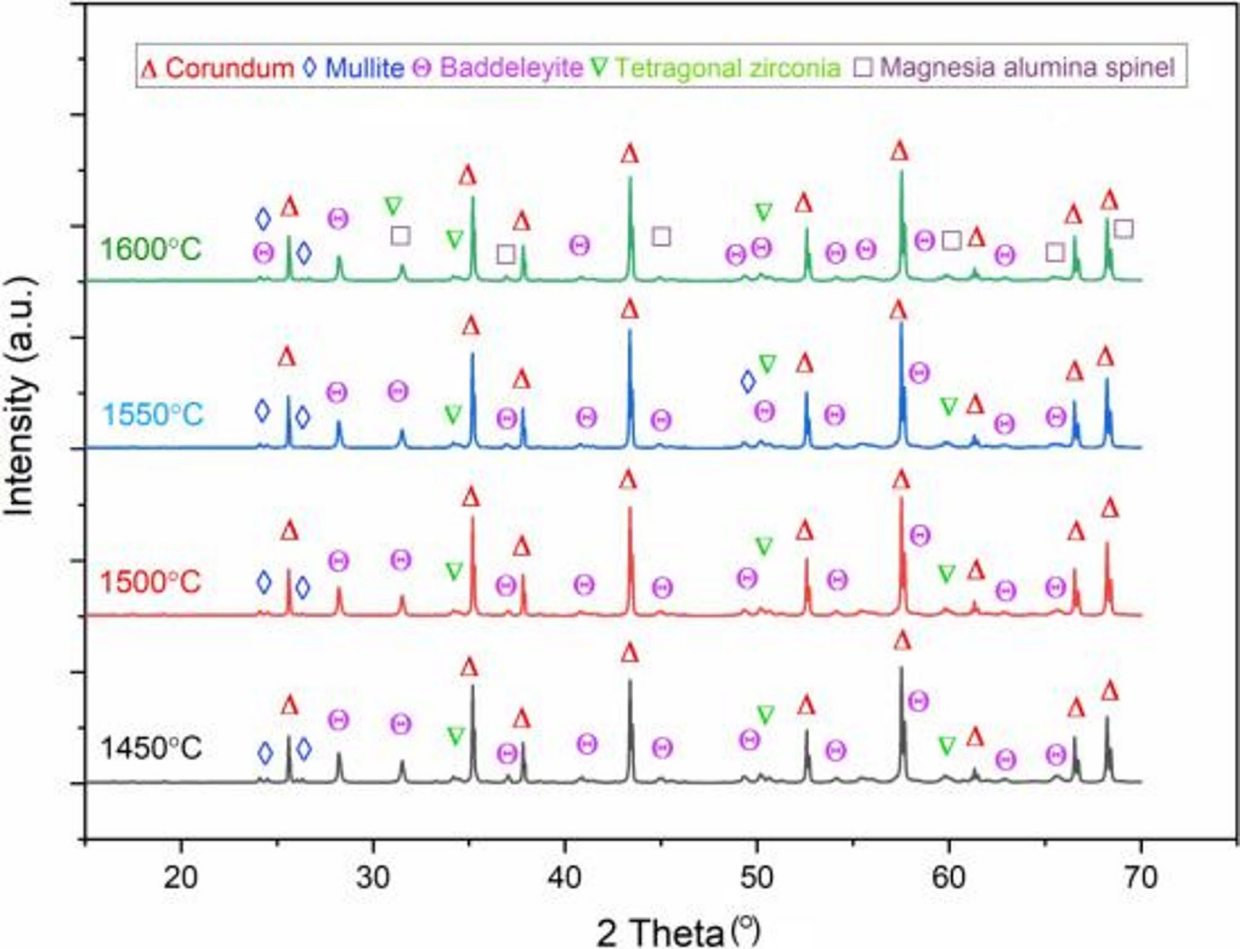
XRD patterns of sintered sample Z15 at different temperatures (1450–1600 °C).

The starting materials for sample preparation were calcined alumina (Al_2_O_3_ ~ 98.20%, with a mean particle size ≤ 10 μm, Alexandria Refractories Company, Egypt), zirconia (ZrO_2_; 99%, particles size ~ 5 μm, Sigma–Aldrich), magnesia (MgO > 95%, particles size < 90 μm, PROLABO), and nano silica (amorphous structure and average particles size ~ 35 nm, Beni Suef University, Egypt^41^. All samples included 80% alumina by weight, which indicates the samples’ ceramic matrix. Magnesia was added to zirconia in a consistent weight% (15 wt%) to stabilize the tetragonal phase at room temperature and as a sintering aid. Actually, 15 wt% MgO may stabilize the tetragonal and cubic phases of zirconia^42^. However, in this study, the ceramic matrix includes alumina and silica in addition to zirconia. We are dealing with a multiphase ceramic matrix. Magnesia can react with these materials. XRD confirmed this, showing the formation of magnesia alumina spinel (Figs. 2 and 3). This indicates that a part of the magnesia stabilized the tetragonal zirconia, whereas the other part was consumed during the formation of the alumina magnesia spinel. As a consequence, 15 weight% was added. Furthermore, the lack of cubic zirconia in the sintered ceramic matrix suggests that the percentage of zirconia is suitable for this investigation. The mixture 1 (Mix 1: zirconia 85 wt% and magnesia 15 wt%) represented the zirconia additions used for this investigation. Zirconia was added at the expense of nano silica at a steady rate of 5 wt% to 20 wt%. Table 1 displays the planned batch composition of the studied samples.

**Table 1:** Chemical composition of batches, wt%.

Sample code	Al_2_O_3_	SiO_2_	Mix 1 (ZrO_2_+ MgO)
Z 0	80	20	0
Z 5	80	15	5
Z 10	80	10	10
Z 15	80	5	15
Z 20	80	0	20
Mix 1: zirconia 85 wt% and magnesia 15 wt%			

### Experimental procedures

Each batch powder was thoroughly mixed using a zirconia ball mill mixing machine for 2 h at a speed of 300 rpm. After thoroughly mixing the powder of each batch, 10 wt% tap water was added to each batch and carefully mixed. The batches were then uniaxially pressed at three different uniaxial compaction pressures of 80, 120, and 160 MPa into cylindrical shapes of 26 mm diameter and 30 mm height, dried at 110 °C for 24 h, and fired for 2 h in an electric furnace at 1450, 1500, 1550, and 1600 °C with a heating and cooling rate of 5 °C/min. Figure 1 displays the preparation method’s process flow diagram.

The Archimedes technique was used to determine the densification parameters for sintered samples, including bulk density and apparent porosity. Apparent porosity is the percentage of a material’s volume composed of open, surface-connected pores (voids) that can absorb fluids, excluding closed or sealed pores. It is calculated as the ratio of open pore volume to the total bulk volume. According to ASTM C20, samples are dried at 105–110 °C to a consistent weight to calculate their dry weight (D). The test samples are then suspended in water and boiled for two hours. They should be immersed in water and not in touch with the container’s hot bottom, i.e. suspended. Heat is turned off, and the samples are suspended in water for at least 12 h. The samples are suspended in water and then weighed to determine their suspended weight (S). Samples are removed from the water and lightly dried using a wet cotton cloth to remove every drop of water from the surface before calculating the saturated weight (W). Bulk Density and apparent porosity can be calculated according to the following equations: Bulk Density (BD) = D/ (W – S) and Apparent Porosity (AP), % = [(W- D)/ (W – S)] × 100.

The linear change (LC) in samples after and before sintering was determined by LC (%) = [(E′ − E)/E] × 100, where E is the diameter of the sample before firing and E′ is the diameter after firing at elevated temperatures. The cold crushing strength (CCS) of all sintered samples was measured using a Matest 24,030 (Matest, Brembate di Sopra, Italy). X-ray diffraction (XRD) analysis was used to identify phase compositions in sintered samples (Bruker D8 ADVANCE with secondary monochromatic beam Cu Kα radiation at 40 kV and 40 mA). The Bruker Company’s EVA program was used to identify phases. Scanning electron microscopy (SEM) of fractured surfaces was used to study the microstructure of selected samples (Quanta FEG 250 with HV 20 kV, magnification up to 5 000 ×, det BSED and WD 13.8 mm). The chemical analysis points were detected using an EDAX unit (AMETEK EDAX Octane Pro) attached to a SEM.

## Results and discussion

### Phase composition

Figure 2 shows the XRD patterns of sintered samples (Z0-Z20) at 1600 °C. Corundum and mullite phases were found to be the major phases in the sample containing 20 wt% nano silica (Z0). In-situ mullite was formed via the solid-state reaction of silica and alumina at sintering temperature: 3Al_2_O_3_ + 2SiO_2_ → 3Al_2_O_3_.2SiO_2_ (approximately 71.8 wt% Al_2_O_3_ and 28.2 wt% SiO_2_). As the amount of nano silica grew from sample Z15 to Z0, in-situ mullite formation increased while residual alumina decreased due to the solid-state reaction; nevertheless, stoichiometric mullite was not reached since the silica addition did not reach the stoichiometric ratio of 28.2 wt%. In contrast, corundum, zirconia (monoclinic and tetragonal phases), and magnesia alumina spinel (MgAl_2_O_4_) phases were observed in the sample containing 20 wt% zirconia. Magnesia alumina spinel phase was formed as a result of the solid-state reaction between alumina and magnesia at sintering temperature: MgO + Al_2_O_3_ → MgAl_2_O_4_. Generally, 15 wt% MgO may stabilize zirconia’s tetragonal and cubic phases^42,43^. However, in this work, we are working with a multiphase ceramic matrix. Magnesia stabilized the tetragonal zirconia, and some of it was consumed by the formation of alumina magnesia spinel. XRD analysis verified this, revealing the formation of magnesia alumina spinel (Figs. 2 and 3). Zirconia phases (monoclinic and tetragonal) are observed on XRD patterns. Furthermore, the XRD patterns show that the sintered ceramic matrix does not contain any cubic zirconia. Corundum, mullite, and zirconia (monoclinic and tetragonal zirconia phases) are the main phases in samples Z5-Z15, with a few diffraction peaks of magnesia alumina spinel (MgAl_2_O_4_). In addition, the intensity of mullite diffraction peaks decreased as the intensity of zirconia and magnesia alumina spinel (MgAl_2_O_4_) peaks increased from sample Z5 to Z15.

Figure 3 shows the XRD patterns of the sintered sample Z15 at various sintering temperatures (1450–1600 °C). The main phases found were the same as those in the sample Z15 sintered at 1600 °C (corundum, mullite, zirconia, and magnesia alumina spinel (MgAl_2_O_4_). Increasing the sintering temperature from 1450 to 1600 °C resulted in enhanced crystallinity of phases. Zirconia was detected in both monoclinic and tetragonal crystalline phases. Tetragonal zirconia (t-zirconia) was detected due to the presence of magnesia, which stabilizes the tetragonal phase of zirconia and causes it to appear at room temperature. However, during the preparation of the sample to powder form for XRD analysis, the tetragonal phase of zirconia (t-zirconia) auto-transformed into the monoclinic phase of zirconia. As a result, monoclinic zirconia was identified using XRD patterns.

### Bulk density and apparent porosity

**Fig. 4:**
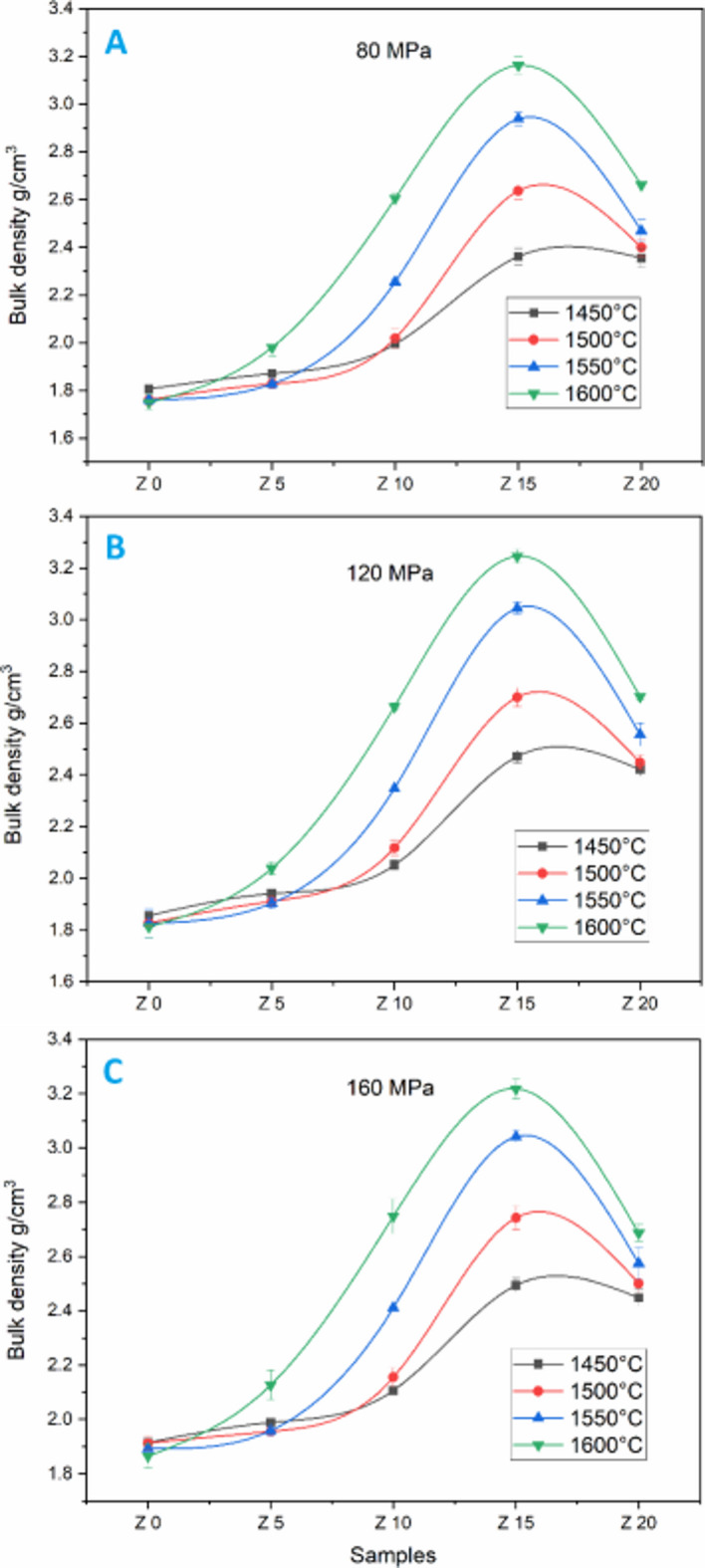
Bulk density (g/cm^3^) of sintered samples at different temperatures (1450–1600 °C) and compaction pressures (80–160 MPa).

Figure 4 shows the bulk density of sintered samples at different temperatures (1450–1600 °C) and compaction pressures (80–160 MPa). At 80 MPa (Fig. 4A), bulk density increased as zirconia content increased in the samples and mullite content decreased from sample Z0 (1.75–1.8 g/cm³) to Z15 (2.36–3.16 g/cm³), then decreased at sample Z20 (2.35–2.66 g/cm³). Zirconia has a greater density (5.70–6.10 g/cm³) compared to mullite (2.7–3.1 g/cm³). Even though sample Z20 had the greatest zirconia percentage, its bulk density dropped. This might be due to the absence of nano silica in the Z20 sample. The specific amount of nano silica addition (5 wt%) plays a crucial impact in sample densification. This nano silica addition is used as a sintering aid to enhance sintering.

As compaction pressures increased from 80 to 120 MPa (Fig. 4B), the bulk density of Z15 at 1600 °C increased (3.16 to 3.22 g/cm^3^). This is due to increased powder compactivity when the compaction pressure increases from 80 MPa to 120 MPa. As a result, the grains became closer together, and the samples’ sintering and densification improved. However, when the compaction pressure increased from 120 to 160 MPa (Fig. 4C), the bulk density remained almost constant (3.22 and 3.24 g/cm^3^), with very little increase in bulk density as the compaction pressure increased from 120 to 160 MPa. This might be due to the powder compact of the samples being close to the maximum value at compaction pressure (120 MPa), and raising the compaction pressure does not have much impact on the densification parameters.

**Fig. 5:**
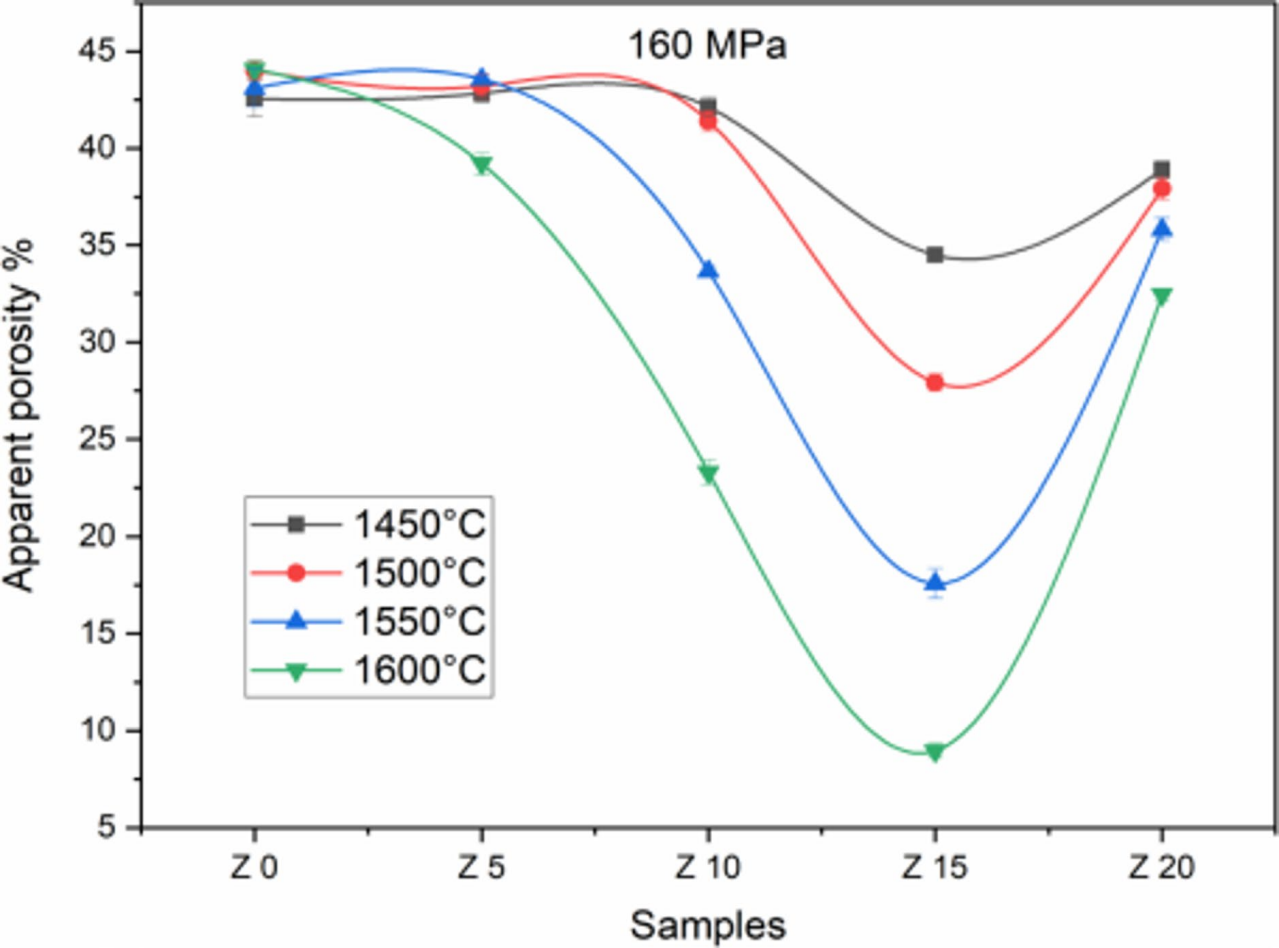
Apparent porosity (%) of sintered samples at different temperatures (1450–1600 °C) and compaction pressure 160 MPa.

Figure 5 shows the apparent porosity (%) of sintered samples at different temperatures (1450–1600 °C) and compaction pressure 160 MPa. The apparent porosity (%) reduced when zirconia additions increased to 15 wt% and sintering temperatures increased to 1600 °C. This is because zirconia fills spaces between alumina and mullite grains in the ceramic matrix, resulting in enhanced densification parameters and lower apparent porosity. Furthermore, increasing the sintering temperature from 1450 to 1600 °C improves the solid-state sintering process by improving particle diffusion in the spaces between grains and closing them. Thus, the apparent porosity of the samples was reduced. The apparent porosity of sample Z15 reduced from 34.49% at 1450 °C to 8.96% at 1600 °C.

**Fig. 6:**
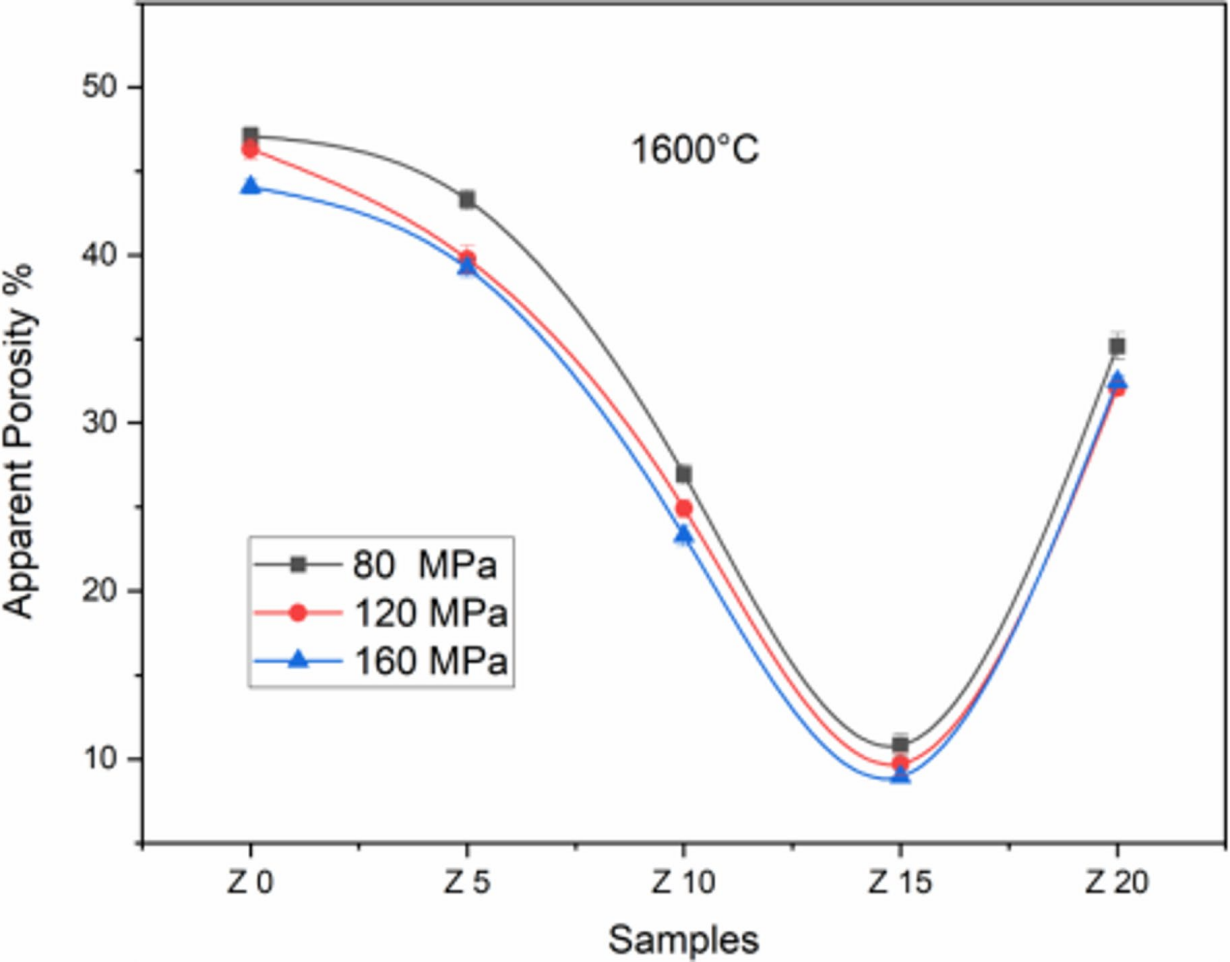
Apparent porosity (%) of sintered samples at 1600 °C and different compaction pressures (80–160 MPa).

Figure 6 shows the apparent porosity (%) of sintered samples at 1600 °C and different compaction pressures (80–160 MPa). Generally, the apparent porosity (%) reduced as the compaction pressure increased from 80 to 160 MPa. This is because raising the compaction pressure improves powder compactness and reduces the spacing between grains. As a result, the ceramic matrix’s sintering improved while its apparent porosity reduced. Nevertheless, we observed that the difference in apparent porosity between compaction pressures 80 and 120 MPa is bigger than the difference between compaction pressures 120 and 160 MPa. That is, when compaction pressures increase over 120 MPa, the impact of compaction pressure on apparent porosity percentage decreases.

### Linear change

**Fig. 7:**
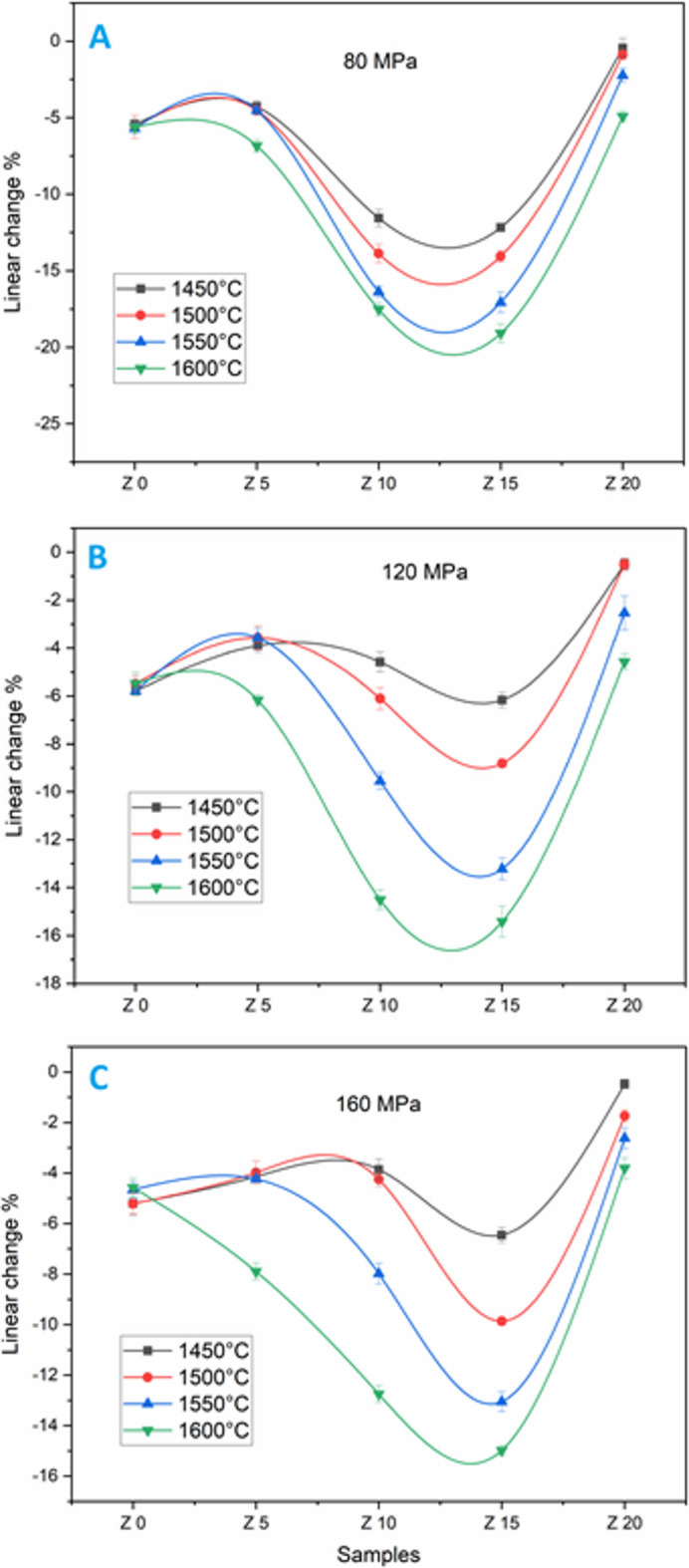
Linear change % in sintered samples at different temperatures (1450–1600 °C) and compaction pressures (80–160 MPa).

Figure 7 shows the linear change % in sintered samples at different temperatures (1450–1600 °C) and compaction pressures (80–160 MPa). All samples demonstrated contraction behaviour. Furthermore, shrinkage increased as the temperature rose from 1450 to 1600 °C. This is due to good sintering in all samples and improved sintering with raising the temperature from 1450 to 1600 °C. However, shrinkage decreased after sintering as the compaction pressure increased from 80 to 160 MPa. This is because when the compaction pressure increased, the space between the grains decreased. 

### Cold crushing strength

**Fig. 8:**
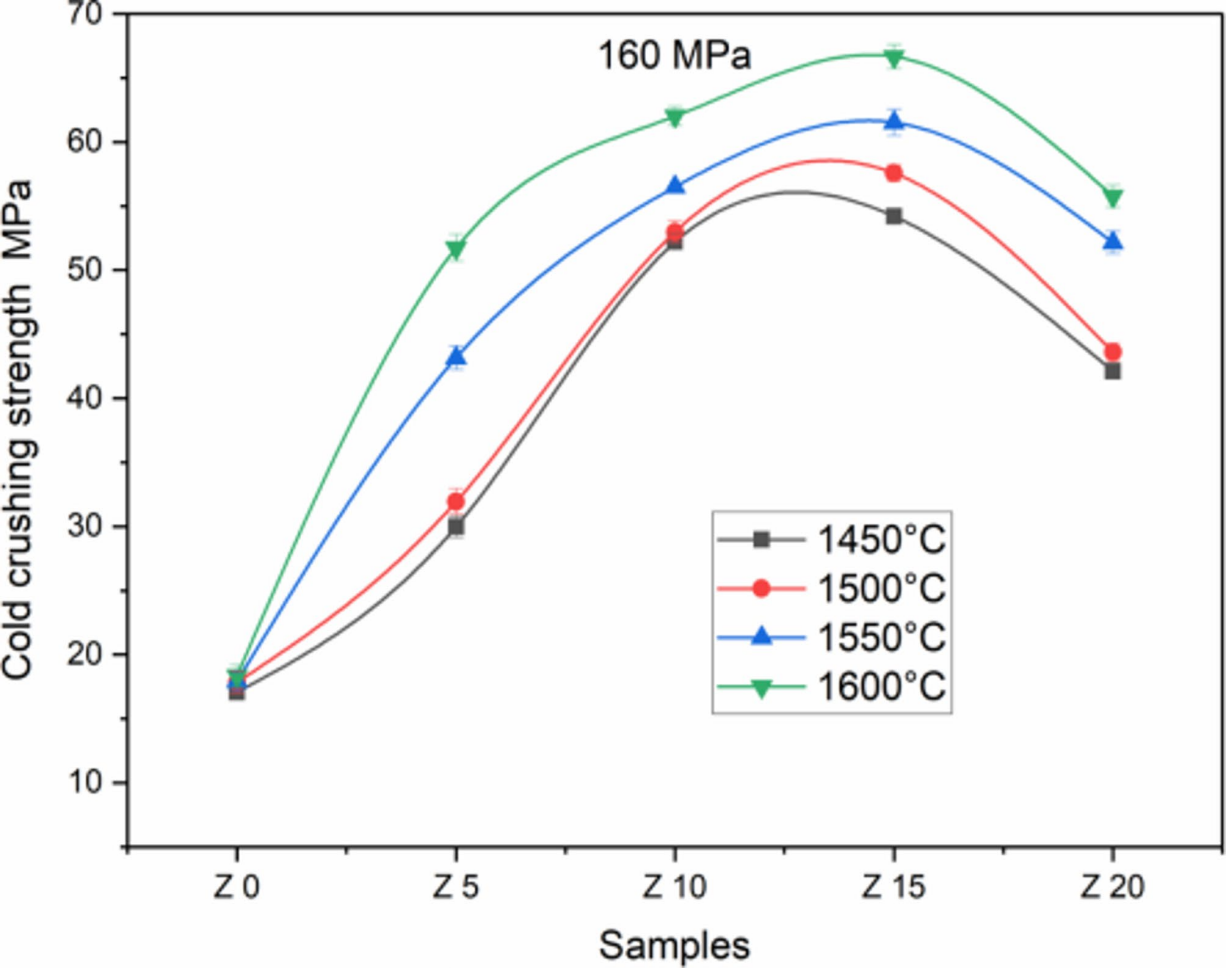
Cold crushing strength of sintered samples at different temperatures (1450–1600 °C) and compaction pressures 160 MPa.

**Fig. 9:**
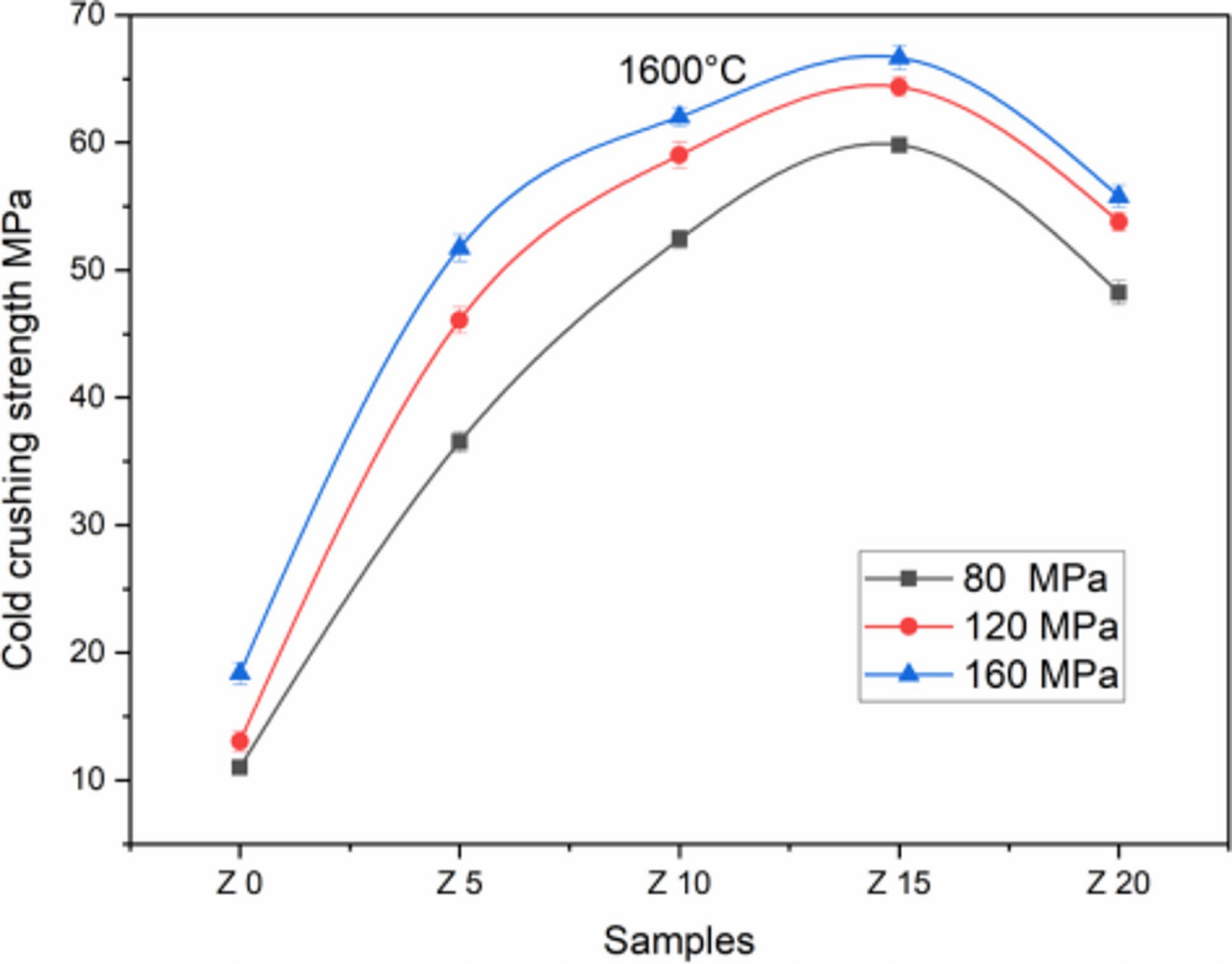
Cold crushing strength of sintered samples at 1600 °C and different compaction pressures (80–160 MPa).

**Fig. 10:**
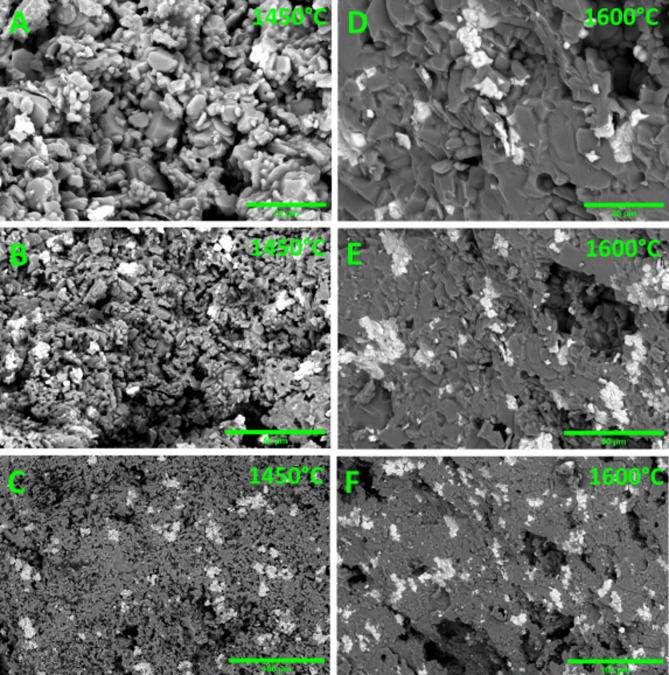
Microphotographs of the chosen sample Z15 sintered at 1450 °C (**A–C**) and 1600 °C (**D–F**).

Figure 8 shows the cold crushing strength of sintered samples at different temperatures (1450–1600 °C) and compaction pressure 160 MPa. The cold crushing strength improved as zirconia content increased from Z0 (17.1–18.37 MPa) to Z15 (54.21–66.66 MPa), and then decreased at Z20 (42.11–55.78 MPa). This is due to mechanically induced tetragonal to monoclinic (t → m) transition in zirconia, which causes volume increase (3–5%) and compressive stress at a crack tip, resulting in crack closure. Furthermore, dispersion toughening caused by energy dissipation occurs when a crack propagates across tougher alumina crystals^8,12^. Thermal expansion mismatches between alumina (α-alumina (8.2 × 10^− 6^ K^− 1^), mullite (4.5 × 10^− 6^ K^− 1^), m-zirconia (6.5 × 10^− 6^ K^− 1^), and t-zirconia (10.5 × 10^− 6^ K^− 1^) generate microcracks^8^. These microcracks absorb energy from the major cracks, reducing crack growth. However, the cold crushing strength of sample Z20 decreased due to the absence of mullite phase. Also, because of the increased porosity in this sample Z20. The porosity has an opposite effect on the mechanical properties of the samples. The cold crushing strength improved when the sintering temperature increased from 1450 ^ο^C to 1600 °C and the compaction pressure increased from 80 to 160 MPa as shown in Figs. 8 and 9. In general, the cold crushing strength of samples is influenced by their porosity and phase composition. A decrease in apparent porosity percentage and an increase in zirconia addition up to 15 wt%, along with 5 wt% nano silica (as a sintering aid), can improve the samples’ cold crushing strength. With raising the sintering temperature from 1450 to 1600 °C and compaction pressure from 80 to 160 MPa, the apparent porosity reduced while cold crushing strength increased. These results may be supported by SEM microstructure (Fig. 10), which shows an increase in porosity at sample Z15 sintered at 1450 ^ο^C than that sintered at 1600 ^ο^C. On the other hand, our results were compared to commercial ones in terms of strength to density, Table 2.

**Table 2:** Density, porosity, and cold crushing strength of some commercial products in comparison with our results.

Category & commercial name	Al_2_O_3_ (%)	ZrO_2_ (%)	SiO_2_ (%)	Bulk density (g/cm^3^)	Apparent porosity (%)	Cold crushing strength (MPa)	Applications according to the manufacturer’s website	Reference
Bonded alumina–zirconia–silica (AZS) with 30% zirconia (ERMOLD)	48	31	18	3.2	13	50	Used in superstructure area requiring high thermal shock resistance and good corrosion resistance such as hot replacement burner blocks, peep-holes and TV blocks	^44^
Sintered zircon–mullite with 20% zirconia (ZMV)	65	18	15	3.1	19	80	Used in forehearth superstructure subject to high corrosion such as sodalime coloring zone or soda-lime extra white glass	^45^
Bonded alumina–zirconia–silica (AZS) with 20% zirconia (ZIRAL 61)	70	19	10	3.15	17	50	Recommended for all applications in feeder expendables, and for superstructures where high corrosion resistance as sodalime glassware, cosmetic glass, high-temperature glass and colored glass	^46^
Sintered alumina–mullite–zirconia with 15% zirconia	80	15	5	3.24	8.96	66.66		Our results

### Microstructure

**Fig. 11:**
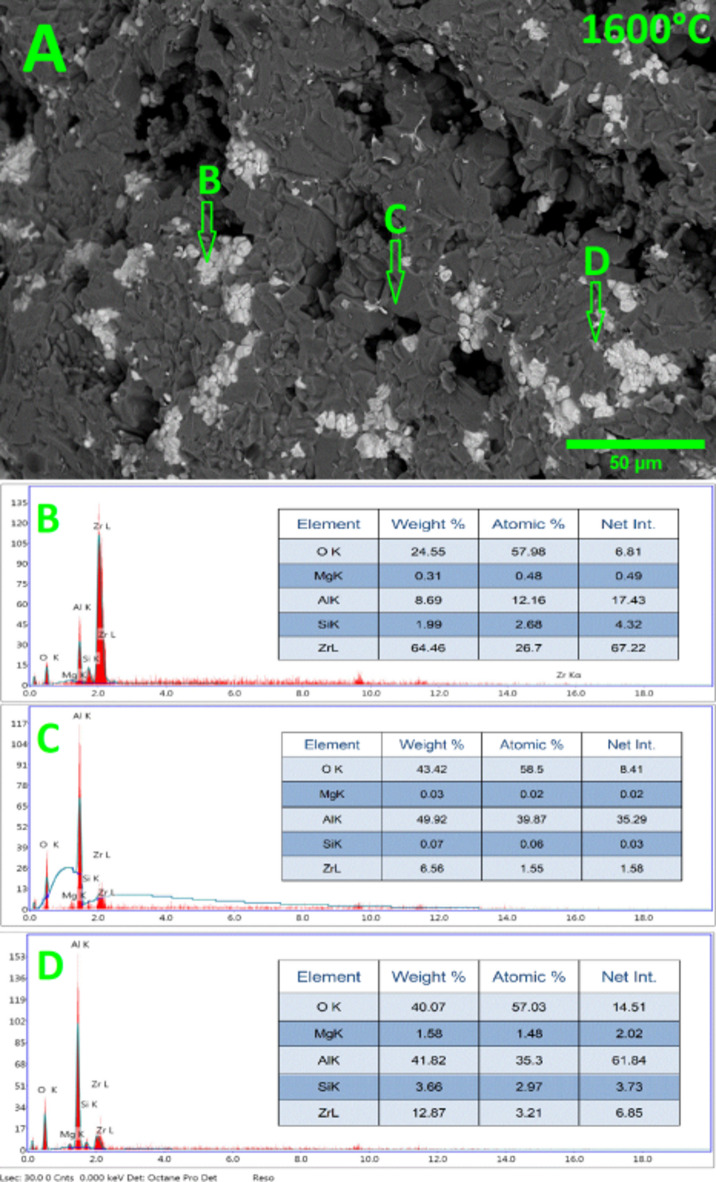
Microphotographs of the chosen sample Z15 sintered at 1600 °C (**A**) and the chemical examination of the solid phases (**B**) Zirconia, (**C**) Alumina, and (**D**) Mullite grains.

Figure 10 shows microphotographs of the selected sample Z15 sintered at 1450 °C (A, B, C) and 1600 °C (D, E, F) at different magnifications. The porosity of sample Z15 sintered at 1450 °C (Fig. 10A, B, and C) increased compared to that sintered at 1600 °C (Fig. 10D, E, and F). The corundum grains had irregular forms and represented the sample’s ceramic matrix. Corundum grains growth faster in sample Z15 sintered at 1600 °C (Fig. 10D) than in sample Z15 sintered at 1450 °C (Fig. 10A). Zirconia appeared as white grains with good dispersion at the grain boundaries of the alumina or mullite matrix in the sample’s microstructure. (Fig. 11B). The inclusion of zirconia pins the alumina grain boundaries^30^. This pinning effect avoids excessive grain coarsening, resulting in a more uniform and refined microstructure, which is essential for maintaining the high strength of the finalised ceramic^26,30,33^. Mullite (Fig. 11D) is difficult to identify in the spaces between the alumina grains (Fig. 11C). This is due to the low amount of nano silica (5 wt%) in the sample. Tables (B, C and D) in Fig. 11 depicted the chemical analysis points of the phases found in the Z15 sample sintered at 1600 °C. The chemical analysis of the solid phases, which contained zirconia grains (point B), alumina grains (point C), and mullite grains (point D), confirmed the phase composition and microstructure identification.

## Conclusions

- The effect of compaction pressure, sintering temperature, and in-situ mullite formation on the sintering and mechanical properties of alumina-mullite-zirconia ceramic was studied.
- At sintering temperatures, in-situ mullite formed due to the solid-state reaction between nano-silica and alumina. The main phases detected are corundum, mullite, and zirconia (monoclinic and tetragonal phases).
- Adding 5 wt% of nano silica improves sintering and densification properties in samples.
- Bulk density increased at 120 MPa compared to 80 MPa. This is due to increased powder compactivity as compaction pressure increases. Grains became closer to each other, sintering and densification of the samples were increased.
- Increasing the sintering temperature from 1450 to 1600 °C improves the solid-state sintering process, causing particles to diffuse between grains and close the grain boundary.
- Cold crushing strength improved as zirconia content increased up to Z15 (15 wt%). This is due to transformation toughening in zirconia grains, which mechanically induces a tetragonal to monoclinic (t → m) transition in zirconia at a crack tip, generating compressive stress and stopping crack propagation. Also, dispersion toughening occurs when a crack propagates across tougher alumina crystals.
- The microstructure of Z15 sintered at 1450 and 1600 °C confirms its phase composition, bulk density, apparent porosity, and cold crushing strength. Corundum grains represented the ceramic matrix, whereas zirconia appeared as white grains with good dispersion at the grain boundaries of the alumina and/or mullite grains in the ceramic matrix. Additionally, as the sintering temperature increased from 1450 °C to 1600 °C, the percentage of porosity decreased and the mechanical properties enhanced.
- Sample Z15 (containing 80 wt% alumina, 15 wt% zirconia, and 5 wt% nano silica) was sintered at 1600 °C and compacted at the pressure 120 MPa. It is suitable for use in refractory and ceramic applications.
- As a consequence, the ideal sintering parameters may be summed by highlighting the crucial importance of 5 wt% nano-silica as a sintering aid, with maximum densification attained at compaction pressure 120 MPa and sintering temperature 1600 °C.

## Data Availability

All data generated or analysed during this study are included in this published article.
